# What should the detection rates of cancers be in breast screening programmes?

**DOI:** 10.1038/sj.bjc.6602345

**Published:** 2005-01-25

**Authors:** S W Duffy, R Gabe

**Affiliations:** 1 Cancer Research UK, Centre for Epidemiology, Mathematics and Statistics, Wolfson Institute of Preventive Medicine, Charterhouse Square, London EC1M 6BQ, UK

**Keywords:** breast cancer, mammography, screening, detection rates

## Abstract

Minimum detection rates at screening are sometimes laid down as standards for breast cancer screening programmes, based on underlying incidence of the disease in the age group screened. Detection rates should also depend on desired sensitivity, mean sojourn time, interscreening interval and the screening round – that is, prevalent (first) or incident (second or subsequent). In this paper, we use these quantities to derive expected, minimum and maximum detection rates proportional to the underlying incidence as well as estimated underlying incidence rates from extrapolation of prescreening trends in England and Wales to derive alternative standard minimum, expected and maximum detection rates per 1000 women screened for the UK Breast Screening Programme, as follows: minimum detection rates should be 4.1 and 4.3 at prevalence screen and incidence screens, respectively; expected rates should be 6.9 and 4.8 and maximum rates of 9.6 and 5.5. These are consistent with observed detection rates in the UK programme.

A frequently used quality standard for mammographic screening is the detection rate, either at prevalence (first) screen or incidence (second or subsequent) screen. Some organisations stipulate an absolute minimum detection rate, others a minimum rate proportional to the underlying incidence (i.e., the incidence one would expect if screening were not taking place).

In the UK, the NHS breast screening programme stipulates a minimum detection rate of invasive tumours of 3.6 per 1000 at prevalence (first) screen and 4 per 1000 at incidence (second or subsequent) screen (NHS Breast Screening Review, 2003). These were estimated using prevalence to incidence ratios from the Swedish Two-county Trial, with the expected interval cancers subtracted to give the incidence screen figure (Moss and Blanks, 1998). The reason the expected rate is higher at the incidence screen is that in the UK programme, which invites women aged 50–64 years (currently being extended to 69 years), average age at prevalence screen is 51 years, whereas average age at incidence screen is 57.5 years, with a correspondingly higher incidence and higher prevalence : incidence ratio. The European Union standard has set a minimum detection rate of 1.5 times the underlying incidence at second and subsequent screens (The European Commission, 2001).

It has been pointed out that the detection rate will depend on the underlying incidence and the interscreening interval (Sarkeala *et al*, in press). The detection rate depends also on the mean sojourn time (MST), the average duration of the preclinical screen-detectable period, and the screening sensitivity. Finally, there is a substantial quantitative difference between prevalence and incidence screening, even if the underlying incidences were equal. This is because a prevalence screen detects (subject to sensitivity) the cancers in the large prevalence pool. For incidence screening, many of these prevalence pool cancers have already been detected at the previous screen, so the detection rate at the incidence screen may be smaller than at the prevalence screen. These considerations are implicit in the calculations of Moss and Blanks (1998), but it is worthwhile to formalise them further, and to avoid explicitly calculating the expected interval cancers.

In this paper, we calculate the expected proportional detection rates for the prevalence and incidence screens for given sensitivity, interval length and MST (or its reciprocal, the rate of progression from preclinical screen-detectable to symptomatic clinical disease). We estimate the underlying incidence rates in England and Wales, and use these in turn to estimate the expected detection rates in the UK programme. Finally, we suggest new minimum and maximum standards for detection rates in the UK programme.

## METHODS

Let *M* represent the mean sojourn time and *S* the screening test sensitivity. Assume a screening interval of *r* years. Let *I* represent the underlying incidence and let *λ*=1/*M* be the rate of transition from preclinical to clinical disease. Paci and Duffy (1991) show that the expected detection rate at prevalence screen is 
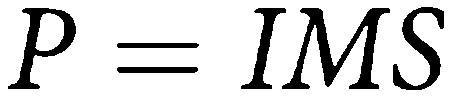


The above rate is for an age group within which incidence and MST are roughly homogeneous. Strictly speaking, *P*, *I*, *M* and *S* are potentially functions of age. This can be seen intuitively since the size of the prevalence pool will be the incidence (*I*) times the average time spent in the prevalence pool (*M*), and the sensitivity will be the proportion of these tumours detectable at first screen. The proportional detection rate will be 
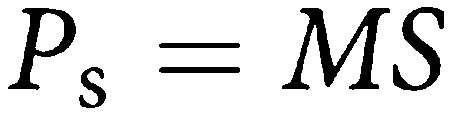


At incidence screens, the expected detection rate is more complicated. Launoy *et al* (Launoy *et al*, 1998; Launoy and Prevost, 2001) show that in steady-state incidence screening, the programme sensitivity, that is, the proportion of tumours diagnosed within the programme that are screen-detected is 
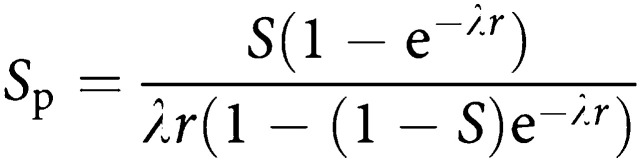


If the expected detection rate of tumours at an incidence screen is *Q*, we have 
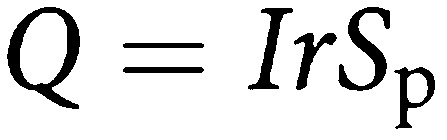


Therefore, 
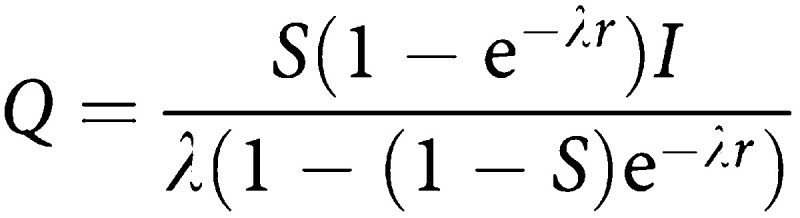


To obtain the proportional detection rate at incidence screens, we divide by *I*

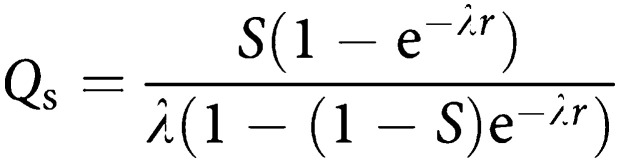


To obtain underlying incidences, we estimated the expected underlying rates for the year 2000 by extrapolation of trends in incidence observed in 1982–1988, before the UK screening programme started. The trends were estimated by Poisson regression (Breslow and Day, 1987), in which the logarithms of the age-specific incidence rates were assumed to increase linearly over calendar time. The reason for the latter approach is to estimate the incidence which would have been obtained had screening not taken place. The screening programme itself is likely to have affected the incidence of breast cancer, notably in the ages of 50–54 years in which most of the prevalence screening takes place.

## RESULTS

### Proportional detection rates

Tabar *et al* (2000) found MSTs of 2.4, 3.7, 4.2 and 4.0 years for the age groups 40–49, 50–59, 60–69 and 70–74 years, respectively. In the age group of 50–64 years, typical estimates of the MST are around 3–4 years (Paci and Duffy, 1991; Tabar *et al*, 2000; Shen and Zelen, 2001) and sensitivity is generally high, in excess of 90%. A 2 : 1 weighted average of the Tabar results for 50–59 and 60–69 years is 3.9 years, which is an estimate appropriate to the age group of 50–64 years. Incidence screens in the UK will on average be in the middle of this range. Prevalence screens, however, will be in the lower 50s, suggesting that a 2 : 1 weighted average of the estimate for 50–59 and 40–49 years age groups is more appropriate for prevalence screens. This is calculated as 3.3 years. With 90% sensitivity, the proportional detection rate at a prevalence screen would therefore be expected to be around 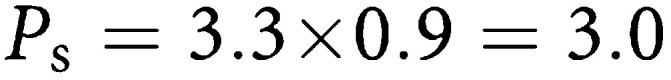


That is, one would expect around three times the underlying incidence at a prevalence screen. At incidence screening, with a 3-year interval, one would expect a proportional detection rate of 
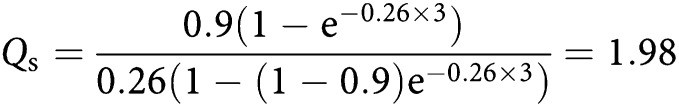
since 0.26=1/3.9. Thus, one would expect approximately double the underlying annual incidence rate at incidence screens. We would not expect these to be mammography standards, since there will be some natural variation around them. A minimum sensitivity of 85% might be reasonable and confidence intervals on estimates of MST suggest that it is unlikely to be lower than 2.1 years for the age at prevalence screen and 3.4 years for the age at incidence screens (Tabar *et al*, 2000). It has also been suggested that upper limits be specified (Sarkeala *et al*, in press) as a possible indicator of overdiagnosis. An MST higher than 4.2 years for age at prevalence screens and 4.6 years for age at incidence screens is unlikely (Tabar *et al*, 2000), and clearly the test sensitivity cannot be higher than 100%. Table 1 therefore gives the expected proportional detection rates (*M*=3.3 or 3.9, *S*=90%), the lower limits (*M*=2.1 or 3.4, *S*=85%) and the upper limits (*M*=4.2 or 4.6, *S*=100%), for prevalence and incidence screens with 1, 2 and 3-year interscreening intervals.

### Absolute detection rates in the UK programme

To obtain detection rates for the UK National Breast Screening Programme, we need estimates of the underlying incidence. Table 2 shows the estimated incidence rates by extrapolation of the prescreening trends in 1982–1988, in 5-year age groups. The average age at incidence screens of 57.5 years suggests that the most suitable estimate of underlying incidence should be that for the ages 55–59 years. If the average age at prevalence screen is 51 years, the appropriate estimated underlying incidence should be a weighted average of the 45–49 and 50–54 year rates, weighting the first by 0.3 and the latter by 0.7.

Table 3 shows the underlying incidences per 100 000 based on the estimated rates, and the corresponding expected, minimum and maximum absolute detection rates per 1000, for prevalence and incidence screens. The incidence screen rates are based on the 3-year interval in the UK programme. This suggests the standard that the prevalence screen detection rates lie between 4.1 and 9.6 per 1000 and the incidence screen rates between 4.3 and 5.5 per 1000.

## DISCUSSION

The stipulation of standards for detection rates should take account the underlying incidence, the screening interval, the MST and the desired test sensitivity. We have derived estimates of proportional detection rates for prevalence and incidence screens based on these, with suggested minima and maxima. In addition, we have applied estimated UK incidence rates to derive standards for the UK National Breast Screening Programme.

One point of caution is that our projected underlying incidence rates differ from the actual observed incidence rates in recent years. The observed incidences in the year 2000 for the four age groups in Table 2 were, respectively, 182.7, 275.2, 284.8 and 311.7 per 100 000. The fact that the estimated rates for ages 50–59 years are lower than observed is desirable, since we require the underlying incidence without the artificial observed increase due to screening. The estimated rates higher than observed for ages 45–49 and 60–64 years, however, suggest that trends in incidence have been more complex than modelled here. The recommended detection rates using the observed rather than estimated incidence rates would be 6.7 at prevalence screen (minimum 5.2–maximum 11.1) and 5.1 at incidence screens (minimum 4.3, maximum 6.3).

We recommend that the prevalence screen detection rates lie in the range 4.1–9.6 per 1000 and incidence screen rates in the range 4.3–5.5 per 1000. The observed rates of 5.4 per 1000 at prevalence and 4.7 per 1000 at incidence screens satisfy both criteria (NHS Breast Screening Review, 2003). We also suggest that three figures might be stipulated in monitoring of screening programmes: a minimum standard, corresponding to our minimum detection rate, a desirable standard to aim at, corresponding to our expected rate, and a maximum, above which further investigation would take place. In the UK programme, the detection rate at prevalence screen is around halfway between our minimum and expected rate, and the rate at incidence screen is very close to our expected.

Our minimum detection rates differ only slightly from existing ones, being around 10% higher. Our expected and maximum detection rates are higher for the prevalence screen than for the incidence screens despite the higher underlying incidence for the latter. This is because the size of the likely pool of occult cases at prevalence screen outweighed the difference in incidence. In practice, a higher rate at prevalence screening is observed in the UK programme (NHS Breast Screening Review, 2003).

It could be argued that our range for the prevalence screen is too wide. This is due to the fact that we have taken conservatively the lower point of the 95% confidence interval on the MST at the ages 40–49 years as the minimum and the upper point of the interval at ages 50–59 years as the maximum (Tabar *et al*, 2000), which is probably conservative due to relative imprecision in the lower age group. Using a formal confidence interval on the weighted average sojourn time of 3.3 would give upper and lower limits for the prevalence screen detection rates of 5.6 and 9.2 per 1000. This is still a rather wide range, due to the uncertainty in estimation of sojourn time at younger ages in which incidence is relatively low. To improve upon this, it would be useful to have large scale interval cancer data from the UK screening programme in order to estimate more precisely the MST in women in their early 50s. Thus, while we believe our methodology is useful and the estimates are reliable, we anticipate that the prevalence screen range could be substantially narrowed with the availability of the appropriate data for precise estimation.

For programmes in other countries, our proportional incidences in Table 1 can be combined with the relevant interscreening intervals and underlying incidences to derive approximate standards. If, however, the age range is different, the likely sensitivity and MST will change. For example, in screening women aged 40–49 years, both MST and sensitivity will be lower.

The UK National Breast Screening Programme is in the process of extending the upper limit to 69 years. When this has been completed, the average age at incidence will be around 60 years. In this case, the appropriate underlying incidence should be the average of the 55–59 and 60–64 year incidences, but the MST would change only very slightly, if at all (Tabar *et al*, 2000). The estimated incidence would be 335.8 per 100 000. This would give a range for the detection rate at incidence screen of 6.0–8.2 per 1000 and an expected rate of 6.7 per 1000.

Our methods depend on several assumptions. We assume a homogeneous exponential distribution of time to progression from preclinical to clinical disease for given age. The exponential distribution assumption is reasonable, although it is possible that there is a mixture of populations, one with a long sojourn time and one with a short. This would lead to a greater overall sojourn time of prevalence screen tumours than incidence screen. The homogeneous model and the exponential distribution have, however, given a reasonable fit to breast screening data in the past (Day and Walter, 1984).

In conclusion, taking account of sojourn time, sensitivity and screening interval, in addition to incidence has yielded new estimates of the likely detection rates in breast screening. The standard minima and maxima suggested by these new rates are consistent with observed rates in the UK programme.

## Figures and Tables

**Table 1:** Expected minimum and maximum proportional detection rates for prevalence and incidence screens, by interscreening interval, assuming a screening age range of 50–64 years

	**Detection rates**		
**Screen (interval)**	**Minimum**	**Expected**	**Maximum**
Prevalence	1.8	3.0	4.2
Incidence (1 year)	0.8	0.9	1.0
Incidence (2 year)	1.4	1.5	1.7
Incidence (3 year)	1.8	2.0	2.3

**Table 2:** Age-specific incidence rates per 100 000 person-years of breast cancer in the year 2000, estimated by extrapolation of prescreening trends

**Age group (years)**	**Incidence estimated**
45–49	213.7
50–54	236.6
55–59	241.0
60–64	430.5

**Table 3:** Observed detection rates in the UK Breast Screening programme, and expected, minimum and maximum detection rates for prevalence and incidence screen, based on incidence estimated by extrapolation from pre-screening trends

**Screen**	**Observed detection rate per 1000**	**Incidence per 10^5^**	**Min rate per 1000**	**Exp rate per 1000**	**Max rate per 1000**
Prevalence	5.4	229.7	4.1	6.9	9.6
Incidence	4.7	241.0	4.3	4.8	5.5
